# Fecal microbiome and metabolome of infants fed bovine MFGM supplemented formula or standard formula with breast-fed infants as reference: a randomized controlled trial

**DOI:** 10.1038/s41598-019-47953-4

**Published:** 2019-08-12

**Authors:** Xuan He, Mariana Parenti, Tove Grip, Bo Lönnerdal, Niklas Timby, Magnus Domellöf, Olle Hernell, Carolyn M. Slupsky

**Affiliations:** 1 Department of Nutrition, University of California Davis, One Shields Ave, Davis, CA 95616 USA; 2 Department of Food Science and Technology, University of California Davis, One Shields Ave, Davis, CA 95616 USA; 3 Department of Clinical Sciences, Pediatrics, Umeå University, SE901 85 Umeå, Sweden

**Keywords:** Metabolomics, Microbiome

## Abstract

Human milk delivers an array of bioactive components that safeguard infant growth and development and maintain healthy gut microbiota. Milk fat globule membrane (MFGM) is a biologically functional fraction of milk increasingly linked to beneficial outcomes in infants through protection from pathogens, modulation of the immune system and improved neurodevelopment. In the present study, we characterized the fecal microbiome and metabolome of infants fed a bovine MFGM supplemented experimental formula (EF) and compared to infants fed standard formula (SF) and a breast-fed reference group. The impact of MFGM on the fecal microbiome was moderate; however, the fecal metabolome of EF-fed infants showed a significant reduction of several metabolites including lactate, succinate, amino acids and their derivatives from that of infants fed SF. Introduction of weaning food with either human milk or infant formula reduces the distinct characteristics of breast-fed- or formula-fed- like infant fecal microbiome and metabolome profiles. Our findings support the hypothesis that higher levels of protein in infant formula and the lack of human milk oligosaccharides promote a shift toward amino acid fermentation in the gut. MFGM may play a role in shaping gut microbial activity and function.

## Introduction

Breastfeeding influences the development of the gut microbiota according to the degree of exclusivity^1,2^. This is at least partly due to the presence of antimicrobial proteins such as secretory immunoglobulin A, lactoferrin and lysozyme in human milk as well as oligosaccharides that function to selectively enhance colonization of specific groups of gut microbes including *Bifidobacterium* spp.^3–5^. Through selection of specific microbes, microbial richness and diversity are ultimately reduced^2^ as they influence the growth of other bacteria^6,7^, which results in modulation of the luminal pH and metabolic profile^8,9^.

Feeding infant formula has been shown to alter the fecal microbiota from that of breast-fed (BF) infants, induce a different serum metabolic profile than in BF infants^10–13^, and has been associated with increased risk of childhood obesity^14,15^. The difference in serum metabolic profiles may in part be due to differences in the intestinal microbiota, but are also likely due to the substantially higher protein concentration of infant formula as compared to human milk. Specifically, formula-fed (FF) infants have been shown to have higher levels of circulating amino acids, amino acid derivatives and urea than BF infants^11,12^.

There is a substantial quantity of nitrogenous compounds from dietary origin that escape digestion, and these compounds are retained in the lumen of the terminal ileum^16^ to provide substrates for microbial fermentation. Amino acids produced by intestinal microbiota are then absorbed and contribute to the host nitrogen pool^17^. Circulating lysine and threonine, that we previously found to be higher in FF infants^13^, are considered metabolites with a gut microbial origin^18–20^, as modification of intestinal microbiota via prebiotic supplementation has been shown to reduce free amino acids in cecum, colon, serum and liver of adult mice^21^, and to slightly lower the blood urea level of infants^22^. These studies support the importance of exploring gut microbiota as contributing factors to infant metabolism.

The milk fat globule membrane (MFGM) has historically been discarded with the milk fat in the manufacturing of bovine milk based infant formula. However, protective activity of MFGM against pathogens and viral infections has been shown^23,24^. Indeed, we found that providing MFGM to young infants in formula reduces the incidence of acute otitis media^25^, and to older infants as a supplement in weaning food reduces the prevalence of diarrhea^26^ and decreases circulating IL-2^27^ as well as trimethylamine-N-oxide^27^, an oxidized microbial byproduct. These studies led to the hypothesis that MFGM may act as a substrate that selectively promotes growth of healthy infant gut microbiota, and that supplementing an MFGM concentrate may alter gut microbial composition and by-products to a profile that is more similar to an exclusively BF reference group.

To provide a mechanistic explanation regarding the influence of MFGM on gut microbiota, we evaluated the fecal microbiome and metabolome of a subgroup of 90 infants who participated in a prospective, double-blind, randomized controlled study^28,29^, where FF infants were randomly assigned to receive a standard infant formula (SF) or a bovine MFGM isolate-supplemented, low-energy, low-protein experimental formula (EF) from ~2 until 6 months of age. An exclusively BF reference group was included in this study. The influence of feeding on the composition and metabolic activity of intestinal microbiota was evaluated through secondary analyses of feces collected from these infants at baseline (~2 months), 4, 6 and 12 months of age via 16S rRNA amplicon sequencing and quantitative metabolomic profiling.

## Results

### Characteristics of study population and data exclusion

Out of a total of 240 infants who participated in a clinical trial concerning the outcomes of feeding a formula supplemented with a bovine MFGM isolate, which initially consisted of BF, n = 80; SF-fed, n = 80, and EF-fed, n = 80 (ClinicalTrials.gov identifier: NCT00624689), following exclusions^28,29^ a subset of 90 infants (15 males and 15 females from each treatment group) were randomly selected for fecal microbiome and metabolome analysis at 2, 4, 6, and 12 months. 96.2%, 96.5% and 82.1% of infants in the BF, SF, and EF group respectively were born vaginally. Inclusion criteria for the original study were: exclusively formula-fed at <2 months of age or intention to exclusively breastfeed until 6 months of age; gestational age at birth: 37–42 weeks; birth weight 2.5–4.5 kg and absence of chronic illness. After data generation, a few samples were further excluded, including infants who stopped consumption of study formula (n = 2), infants who had no record of a food diary (n = 1), infants from the BF group that were heavily mixed-fed (n = 3), infants from the FF group who consumed another formula (n = 4), and infants who had a record of antibiotics use (n = 3). For fecal metabolome data, samples that contained urea were suspected of being contaminated by urine since normal fecal samples do not contain urea due to bacterial urease activity. Therefore, fecal samples containing urea were excluded from analysis. The number of samples from each intervention and time point are summarized in SI Tables 1, 2.

### Study formula intake and complementary food consumption

Our previous work on this cohort showed that infants consuming the low-energy, low-protein EF consumed a higher volume, yielding the same energy intake as infants consuming SF^28^. In this randomized subgroup, the intake of formula was only slightly higher in the EF group (effect sizes |δ| at each month were negligible or small, SI Fig. 1a). Although EF contains less energy than SF (SI Table 3), there was no significant difference in energy intake from consumption of these two formulas (Kruskal-Wallis’ H test, effect sizes |δ| at each month were negligible or small, SI Fig. 1c). Starting from 4 months, a variety of complementary foods were introduced (SI Fig. 1b) as suggested by the Swedish National Food Agency recommendation^30^. With increasing energy from complementary food, energy from study formula decreased between 4 and 6 months of age (SI Fig. 1c). By 6 months, fewer than 30 infants in this subgroup were still exclusively BF or fed study formula and had no exposure to complementary food (SI Fig. 1b,d). To account for the influence of complementary food introduction in our analysis, samples collected at 4 and 6 months were divided into two subgroups depending on complementary food intake and defined as: with (>10% daily energy from) or without (<10% daily energy from) complementary food.

### Gut microbiome

**Figure 1:**
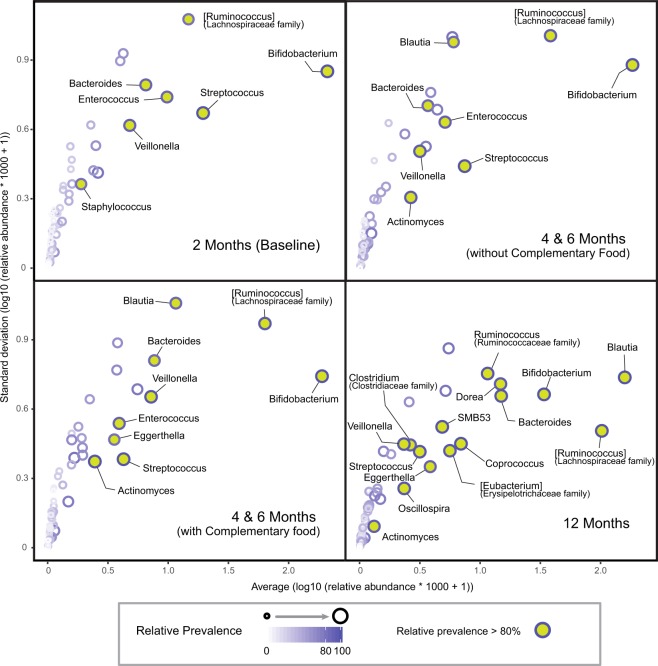
The core fecal microbiota of infants. The core microbiota is defined as highly prevalent microbes at the genus level present in >80% of samples.

To explore the effect of early diet on intestinal microbiota, 16 s rRNA gene sequencing of infant feces was performed. Analysis of core microbes of all infants revealed a high prevalence of *Bifidobacterium*, *Streptococcus*, [Ruminococcus] (from Lachnospiraceae family), *Veillonella*, *Enterococcus* and *Bacteroides* from 2 to 6 months of age. Over time, as more weaning food was introduced, the relative abundance of *Blautia* in the stool increased (Fig. 1, SI Fig. 2). By 12 months of age, *Bifidobacterium*, *Streptococcus* and *Bacteroides* remained highly prevalent. With increases in microbial richness, additional microbes become highly prevalent in the gut (Fig. 1).

**Figure 2:**
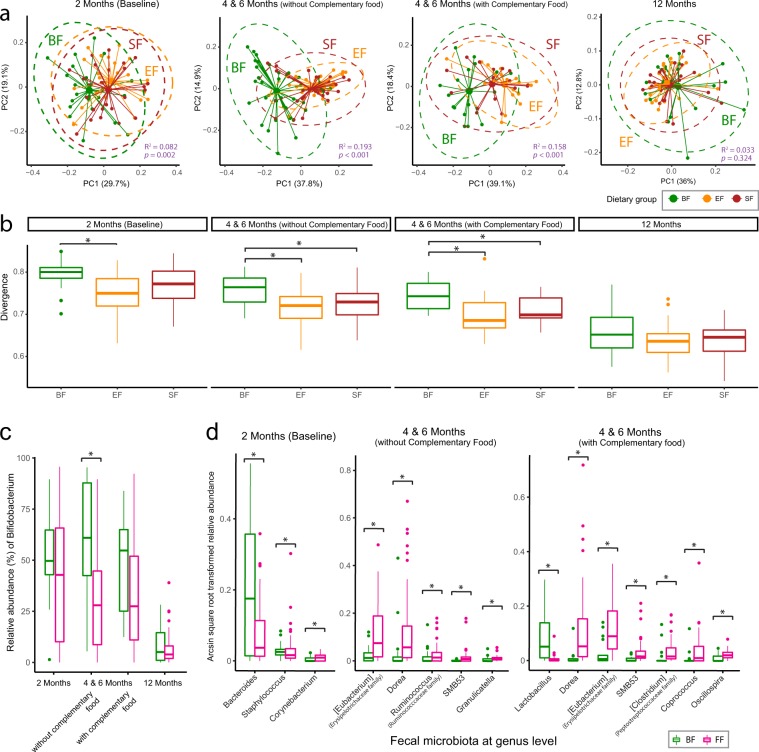
Analysis of the community structure of fecal microbiota reveals differences between breast-fed (BF, green) and formula-fed (Experimental Formula, EF, orange; Standard Formula, SF, red) infants at 2, 4 and 6 months of age. (**a)** Principal Coordinates Analysis (PCoA) of the log transformed weighted Unifrac distance. The centroids of each cluster (centroid of mass) are calculated as the average of PC1 and PC2 for each group. The ellipses were constructed based on multivariate normal distribution at 95% confidence level. The effect size (R^2^) and significance (p-value) between dietary groups were evaluated using permutational MANOVA via the Adonis test (permutation = 999). (**b**) Divergence (the spread within the group) was significantly higher in the BF infants during the exclusive feeding period. The measurement of divergence is calculated as 1- the average spearman correlation between samples and the overall group-wise average. The group difference was evaluated using a Kruskal-Wallis H test follow by the post-hoc Dunn test at p < 0.05. (**c,d)** Significantly differentiating intestinal microbes between the breast-fed (BF, green) and formula-fed (FF, pink) infants. The group differences were evaluated using Analysis of Composition of Microbiomes (ANCOM) followed by FDR correction at p < 0.05.

As expected, higher levels of fecal *Streptococcus* was observed at 2 months of age (SI Fig. 2), which is likely due to bacterial communities arising from the environment, areolar skin and mother’s milk^31–33^. At 4 and 6 months of age when no or low complementary food was introduced, the impact of feeding on the fecal microbial profile was the largest (Fig. 2a), with higher abundance of *Bifidobacterium* species in BF compared with FF infants (Fig. 2c). BF infants had a more heterogeneous fecal microbiome profile only during the exclusive feeding period (Fig. 2b). By 12 months of age, the fecal microbiome became more homogenous (Fig. 2b) and the BF and FF fecal microbiomes became indistinguishable (Fig. 2a).

While fecal *Bifidobacteria* (Fig. 2c) and other lower abundant microbes (Fig. 2d) were significantly different between BF and FF, the difference between the two FF groups was minor. Some infants who consumed EF had a higher percentage of *Akkermansia* in the stool, and this was more prevalent when no or low complementary food was introduced (SI Fig. 3a). At 12 months, the number of individuals with *Haemophilus* was lower in infants who consumed EF compared with those who consumed SF (SI Fig. 3b, ANCOM test followed by FDR correction).

In comparison to BF infants, FF infants showed a slightly higher microbial richness (observed species) at 2 months (p = 0.077, Kruskal Wallis H test), but not statistically different (or close to statistically different) at other time points examined. When applying “Tail” statistics index (a more robust estimate of microbial diversity to account for low-abundance rare species^34^), no significant difference was detected between the two FF groups or between BF and FF infants, which may be due to the fact that some of the FF-infants were likely breast-fed after delivery for some time before enrolment at ~2 months of age.

### Fecal metabolome

**Figure 3:**
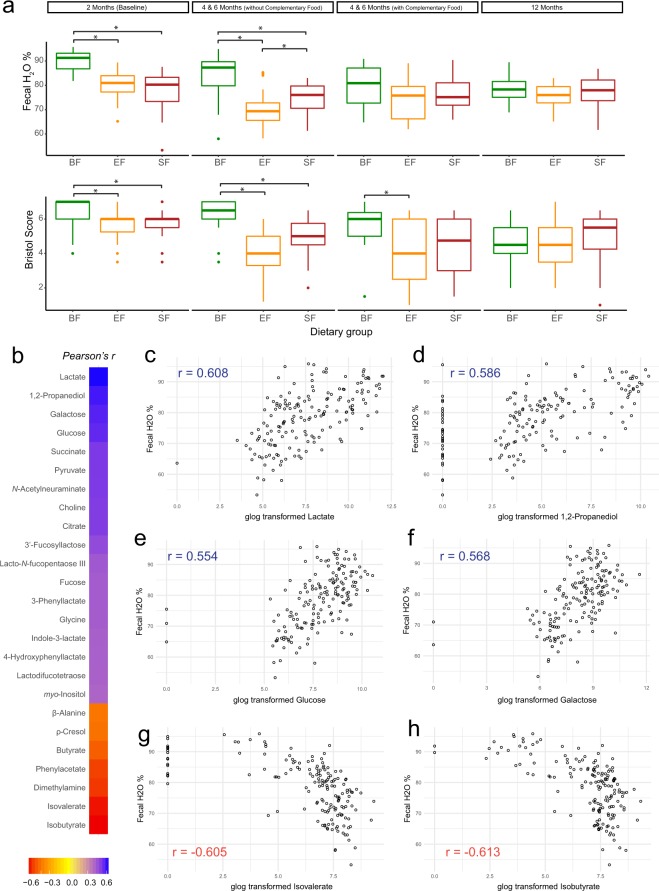
(**a**) Fecal water % and Bristol score are significantly higher in the breast-fed (BF) infants during the exclusive feeding period. The group difference was evaluated using Kruskal-Wallis H test followed by a post-hoc Dunn test, p < 0.05. Fecal metabolite concentrations are associated with fecal water %. (**b**) Heatmap of the correlation coefficient (Pearson r > 0.4 or <−0.4, p < 0.05) of fecal metabolite concentrations correspond to fecal water %. Amount of water in infant stool is positively correlated with fecal concentration of (**c**) lactate, (**d) 1,2-propanediol**, (**e**) glucose and (**f**) galactose and is negatively correlated with (**g**) isovalerate and (**h**) isobutyrate. The correlation between fecal water % and metabolite concentration were evaluated using data collected from 2 months (baseline) and 4 and 6 months (without complementary food).

To evaluate the impact of early diet on intestinal microbial fermentation capability, corresponding fecal metabolites were examined. In agreement with prior studies^35^, BF infants in this cohort also had higher stool frequency and softer/looser stool consistency compared to FF infants^25^. In this subgroup, we observed significantly higher fecal water % and Bristol score in BF infants compared to the FF infants when infants were exclusively breast-fed or formula-fed respectively. However, after introduction of complementary food the water content in the stool of BF infants decreased to a level similar to both formula groups (Fig. 3a). Comparison of the water content in the stool of the formula groups revealed that infants consuming EF had less water in their stool than infants consuming SF (Fig. 3a). Since fecal water % was significantly different between groups, instead of normalizing fecal metabolite concentration by wet or dry fecal weight, values were expressed in µM.

Fecal metabolite concentrations were associated with the amount of water in the stool. With increased water, higher concentrations of sugar monomers (glucose, galactose) and fermentation byproducts from carbohydrate metabolism (lactate, 1,2-propanediol, and succinate) were observed. In contrast, the concentration of microbial by-products from amino acid degradation (isobutyrate, isovalerate, dimethylamine, and phenylacetate) were associated with lower fecal water % (Fig. 3b–h).

**Figure 4:**
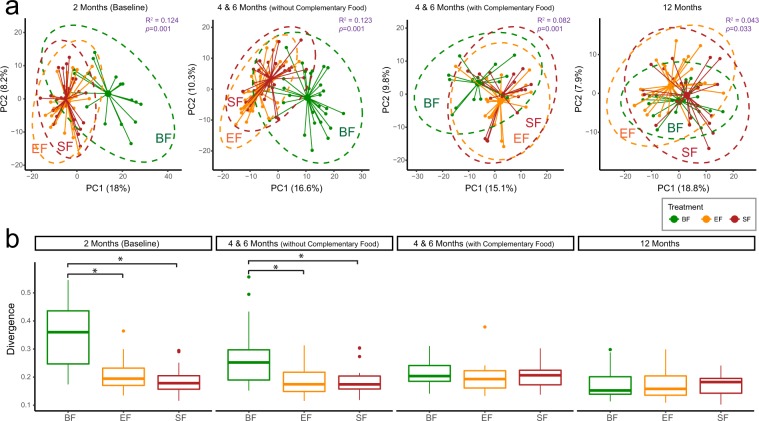
The fecal metabolome reveals differences between breast-fed (BF, green) and formula-fed (Experimental Formula, EF, orange; Standard Formula, SF, red) infants at 2, and 4 & 6 months of age. (**a**) Principal Coordinates Analysis (PCoA) of the Euclidean distance matrix of the generalized log transformed fecal metabolite concentration data. The centroids of each cluster (centroid of mass) were calculated as the average PC1 and PC2 of all samples for each group. The ellipses were constructed based on multivariate normal distribution at 95% confidence level. The effect size (R^2^) and significance (p-value) between dietary groups were evaluated using permutational MANOVA via the Adonis test (permutation = 999). (**b)** Divergence (the spread within each group) is significantly higher in BF infants during the exclusive feeding period. The measurement of divergence was calculated as 1- the average spearman correlation between samples and the overall group-wise average. The group difference was evaluated using Kruskal-Wallis H test follow by post-hoc Dunn test, p < 0.05.

Starting from 2 months of age, a profound difference in the fecal metabolome between BF and FF was observed and continued to 4 and 6 months when no or small amounts of complementary food were introduced. The effect of diet on the fecal metabolome was reduced in the group with complementary food consumption and became indistinguishable at 12 months of age (Fig. 4a, Adonis Test). In agreement with the fecal microbiome data (Fig. 2b), BF infants had a more heterogeneous fecal metabolome profile than FF infants, while the difference between the two FF groups was almost indistinguishable through Principal Coordinates Analysis (Fig. 4b).

**Figure 5:**
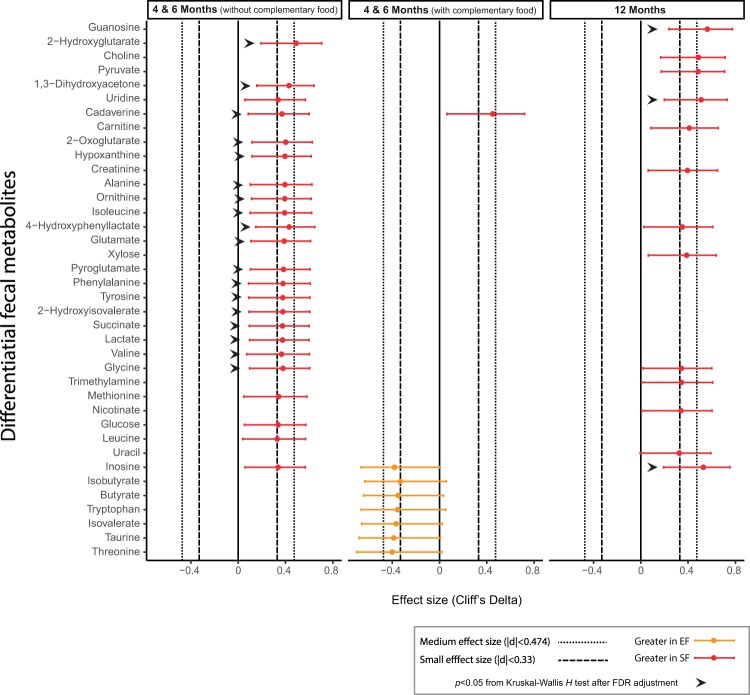
Fecal metabolites that are significantly different between formula-fed infants who consumed standard formula (SF, red) and experimental formula (EF, orange).

The major difference between the two FF groups was revealed during the exclusive feeding period, with lower levels of amino acids (ornithine, isoleucine, glutamate, phenylalanine, tyrosine, valine and glycine), and 2-hydroxyisovalerate (branched-chain amino acid degradation product), cadaverine (lysine breakdown product), and 4-hydroxyphenyllactate (tyrosine breakdown product) in the EF group (Fig. 5, Cliff’s Delta, Kruskal Wallis H test). Lactate and succinate, the two fecal metabolite markers that are positively correlated with amount of water in stool (Fig. 3b,c), were also lower in EF group (Fig. 5), and corresponded to the lower fecal water % (Fig. 3a). Intermediate metabolites from purine degradation metabolism, including hypoxanthine, were significantly lower, and inosine tended to be lower in the EF group. Although glycosylation of MFGM protein has been reported^36^, supplementing a bovine MFGM concentrate to the EF did not drive the fecal metabolome to the extent expected for human oligosaccharides (HMOs), thus the differences observed were likely derived from other active ingredients.

In general, acetate was the most dominant metabolite in the stool (SI Fig. 4). The appearance of larger and more diverse fecal sugar monomers are unique markers of breastfeeding, and include *N*-acetylglucosamine, fucose, galactose and glucose. In comparison, the major sugar monomers in the stool of FF infants were mostly galactose and glucose, sugar monomers from lactose. By 12 months of age, glucose was the major sugar monomer in infant stool. Only a few BF infants at 2 months of age had high levels of detectable HMOs in the stool, and the majority of these infants had lower concentrations of by-products from complex carbohydrate degradation (SI Fig. 4).

**Figure 6:**
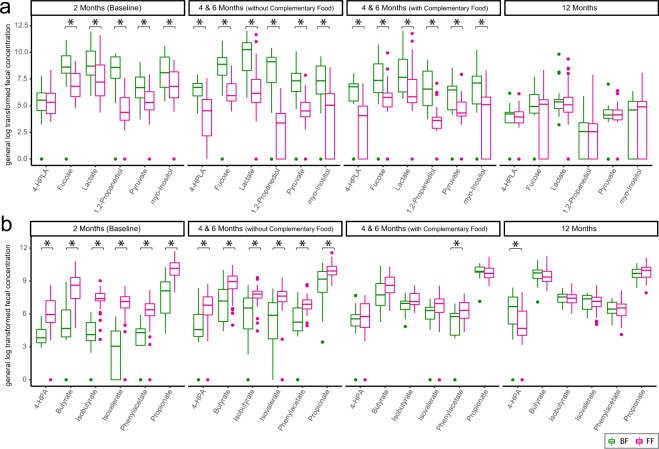
Significantly differentiating fecal metabolites that are (**a)** higher and (**b**) lower in breast-fed (BF, green) than formula-fed (FF, red) infants. The group differences were evaluated using Kruskal-Wallis H test by FDR correction at p < 0.05. The complete list of differentiating metabolites is in SI Fig. 5. Abbreviation: 4-HPLA: 4-hydroxyphenyllactate, 4-HPA: 4-hydroxyphenylacetate.

As breastfeeding continued, although more complementary food was introduced, the signature of breastfeeding persisted in the stool. BF infants exhibited higher levels of 4-hydroxyphenyllactate, fucose, lactate, 1,2-propanediol, pyruvate, and *myo*-inositol than the FF infants (Fig. 6a). In contrast, the FF-specific metabolic signature, included higher levels of butyrate, propionate, isobutyrate, isovalerate, phenylacetate and 4-hydroxyphenylacetate, which were no longer significant when more “carbohydrate-rich” complementary foods were introduced (Fig. 6b, Kruskal-Wallis H test). The complete list of differential metabolites between BF and FF stool is present in SI Fig. 5.

## Discussion

Intestinal microbiota adapts and responds to the availability and distribution of fermentable substrates delivered from food. After escape from digestion, lactose and complex carbohydrates (including free HMOs, protein-derived and host-derived glycans) become substrates for gut microbial fermentation. Introduction of complementary food to either BF or FF infants disrupts the BF- or FF- specific fecal profile (Figs 2a, 4a). As weaning begins, the infant gut microbiota is exposed for the first time to a new range of nutrients, and as a result, inter-individual variation becomes less pronounced and the composition starts to resemble that of an adult-like microbiota. The abundance of *Bifidobacterium*, the predominant genus of the gut microbiota of BF infants, was no longer significantly higher than in FF infants following introduction of complementary food (Fig. 2c). However, before 6 months of age, if breastfeeding continued, complementary food introduction had a negligible effect on the key BF-specific fecal metabolite markers (Fig. 6a).

In this study, a bovine MFGM concentrate was supplemented to infant formula as an attempt to narrow the metabolic and microbial gap between BF and FF infants. Although some of the MFGM proteins are glycosylated^36^, which could provide a substrate for microbial fermentation, the amount of bovine MFGM supplemented in the current study is lower (approximately 0.48 g/L) compared to a recent study on rat pups (1.2 g/L) that showed a significant change in colon microbial composition and diversity with MFGM supplementation^37^. In comparison to the difference in the serum metabolome and other biochemical, clinical and cognitive measurements, the influence of MFGM supplementation on the overall oral microbiota^38^ and fecal microbiota (Figs 2a, 4a) was moderate and did not override the effect of formula, as infants who consumed MFGM-supplemented formula were more similar to those who consumed SF than those who were breast-fed. However, during the exclusive feeding period, lower concentrations of a few metabolites were observed in the EF in compared to the SF group (Fig. 5), providing a complementary view toward a potential functional change in the gut microbial community. Fecal hypoxanthine was lower in the EF-fed infants (Fig. 5), which is in line with the presence of hypoxanthine dehydrogenase/oxidase in bovine MFGM^39^. The reduced level of fecal hypoxanthine, together with the high abundance of *Akkemansia* in a few of the EF-fed infants (SI Fig. 3a), should be further confirmed in a larger clinical study. At 12 months of age, the incidence of having a high prevalence of fecal *Haemophilus*, a genus consisting of several pathogens, was lower in EF-fed infants (SI Fig. 3b). This observation suggests a potential long term impact of MFGM supplementation on the fecal microbiota that has not been reported elsewhere, and builds on previous studies in this cohort showing a lower incidence of acute otitis media in EF-fed infants at 6 months of age^25^. Further studies with larger sample sizes and targeted pathogen detection are needed to confirm these observations.

In comparison to formula-feeding, human milk consumption yielded higher levels of lactate, pyruvate, 1,2-propanediol, *myo*-inositol, fucose and 4-hydroxyphenyllactate in the stool (Fig. 6a). The large amounts of lactate in BF infant stool reported here and elsewhere^40^, are expected to be produced by *Bifidobacterium*, *Bacteroides, Enterococcus* and *Streptococcus*^41,42^ that are also primary colonizers of the infant gut (Fig. 1). However, in comparison to other bacterial genera, the metabolic capability of the species belonging to the *Bifidobacteria* genus is highly conserved with enriched saccharolytic modules in their genomes that are specialized for efficient HMO utilization^43^. Several *Bifidobacteria* subspecies (including *B. longum* subsp. *infantis*^44^, *B. longum* subsp. *suis BSM11-5*^45^, *B. kashiwanohense*^45^, and *B. bifidium*^46^) harbor α-fucosidases and demonstrate the ability to produce 1,2-propanediol from fucose and fucosyllactose^45^. *Bifidobacterium* has been shown to produce a considerable amount of 4-hydroxyphenyllactate *in vitro*^47^, providing an explanation for the high 4-hydroxyphenyllactate found in the stool of BF infants.

While breastfeeding-derived fecal metabolic markers are associated with the utilization of carbohydrates, FF induces higher levels of branched chain fatty acids (BCFAs, isobutyrate and isovalerate) and phenylacetate (Fig. 6b), which are microbial end-products of peptide and amino acid fermentation. These have been shown to be elevated in *in vitro* incubation of intestinal content^48,49^, colon content from rodents, and in stool from individuals consuming high protein diets^50–52^. The higher fecal levels of propionate and butyrate in FF infants (Fig. 6b and elsewhere^53,54^) may be due to degradation of certain amino acids^55^ or production by species from the *Eubacterium* genus (Fig. 2d), which demonstrate an ability to utilize lactate and produce butyrate and propionate^56,57^.

In accordance with the higher protein intake provided by formula, stool ammonia has been reported to be significantly higher in exclusively FF infants compared to their BF counterparts^58^ which can be explained by excess dietary protein that escapes digestion^59^. This further implies that the intestinal microbiota in FF infants is likely to participate in significant catabolism of amino acids in the intestinal lumen. To an extent, the presence of ammonia reduces the uptake and utilization of short chain fatty acids (SCFAs) by colonocytes, and subsequently decreases the production of ketone bodies and CO_2_ ^50,60–62^; therefore, the elevated butyrate level in the stool of FF infants (Fig. 6b) could also, in part, be due to reduced uptake by the colonocytes. Furthermore, ammonia can be easily absorbed by colonocytes, enter the circulation, and be detoxified to urea in the liver. This may contribute to the high urea concentration observed in the serum metabolome of FF infants^13^.

Infant formula contains considerably more protein than human milk^63^. A high protein content in the diet not only provides substrates for amino acid degradation, but may also induce protease activity in the colon^50^. In addition, proteases are more active in the neutral to alkaline pH range^64^. From the proximal to the distal colon, intestinal luminal pH^65^ and protease activity^66,67^ progressively increases as the amount of SCFA declines^65,68^. Gut microbial carbohydrate fermentation is also higher in the proximal colon^69^, whereas protein fermentation mostly takes place in the distal colon^67^. We speculate that carbohydrate fermentation in the proximal colon influences protein fermentation in the distal colon. When a substantial amount of HMOs reach the colon, rapid microbial fermentation is induced. As high total acids are produced from complex carbohydrate fermentation, intestinal bicarbonate buffering capacity is insufficient, and thus induces significant acidification of the lumen^70,71^. It has been shown that BF infants with greater utilization of HMOs exhibit a lower stool pH^72^, and rats consuming inulin alone or with *Bifidobacterium longum* have significantly reduced caecal pH and ammonia concentration^73^. This further supports the observation that acidic pH can decrease microbial uptake of most amino acids, reduce the rate of amino acid fermentation and subsequently decrease the net production of ammonia and BCFA^48^.

Since the amount of oligosaccharides is low in infant formula, the lack of complex carbohydrate substrates from the diet results in a higher stool pH in FF infants compared to BF infants^40,54^. In contrast, incorporating galacto-oligosaccharides and fructo-oligosaccharides in infant formula reduces the stool pH to a level more similar to BF infants^53,54,74–76^. Interestingly, none of the key metabolites that are different between BF and FF infants (Fig. 6) were observed in our previous work when comparing BF and FF rhesus monkeys^77^. Rhesus monkey milk contains lower oligosaccharide concentrations than human milk^78,79^. Therefore, the difference in fecal metabolome of BF and FF human infants observed here could be largely explained by the availability of complex carbohydrates in the diet for microbial utilization.

During the exclusive feeding period, FF infants had less water content in the stool (Fig. 3a). In addition, a harder stool and a longer intestinal transit time have been associated with carbohydrate deprivation and a more extensive proteinaceous substrate breakdown^80,81^. Dietary inclusion of fermentable complex carbohydrates has been shown to be a promising approach to reduce the rate of microbial protein fermentation^82–84^. Indeed, infants who consumed formula containing non-digestible oligosaccharides (fructo-oligosaccharides, galacto-oligosaccharides) had faster transit times and softer and more frequent stools^22,74–76,85–87^. Here, we observed that a watery stool is associated with an excess amount of sugar monomers and by-products from oligosaccharide fermentation (Fig. 3b–f). These microbial-derived products, if not utilized by the host or other microbes, may also act as organic osmolytes, drawing water from the epithelium into the lumen, leading to an even softer stool.

One strength of the present study is the double-blind randomized design among FF infants. Comparisons between FF and BF infants in the present study should be made with caution since these two groups were not randomized and potential confounding factors involving feeding choice cannot be ruled out. Another limitation of this study is the lack of information on the volume of human milk consumed and mother’s milk composition. It is well established that the composition of HMOs is driven by maternal secretor status and Lewis Blood group status^88,89^. Furthermore, by 12 months of age, only 6 infants in the BF group had a record of breast milk consumption. Although Bäckhed *et al*. have shown that the influence of breastfeeding on the gut microbiome remains at 12 months if breastfeeding persists^1^, we were not able to confirm this in the present study. Additionally, a large number of infants in all dietary groups consumed probiotics (containing *Lactobacillus*) during the study intervention period. Although the relative abundance of *Lactobacillus* in stool was low (<3%), we cannot rule out its potential contribution to the infant fecal metabolome^90^.

## Conclusions

The results obtained in the present study clearly show the importance of early life nutrition on the community profile and functional characterization of intestinal microbiota. Breastmilk or formula-feeding as well as complementary food influence gut microbiota and the luminal environment. Building on previous work^13,25,28,29,38,91^ on this cohort, this study provides information on the effect of feeding on gut microbiota in the first year of life. The different fecal microbiomes are reflected by differences in fecal water % and concentration of microbial by-products in the stool. With limited oligosaccharides present and high protein levels in infant formula, we speculate that the difference observed between the BF and FF infant fecal microbiome and metabolome can be explained by an alteration in the availability and microbial utilization of carbohydrates and proteins as energy substrates. The difference between the fecal microbial taxonomic profiles of MFGM supplemented EF and SF was moderate in comparison to the distinct difference between BF and FF infants. However, an influence of MFGM on the fecal metabolome was observed, and may be associated with a change in microbial activity and function. Further studies are needed to explore the linkage between the potential antimicrobial property of MFGM proteins and the specific alteration we observed in the fecal metabolome.

## Methods

### Study population

This double-blinded, parallel randomized controlled trial took place at Umeå University Hospital, Umeå, Sweden after approval by the Regional Ethical Review Board in Umeå (Dnr 07-083 M) for all experimental protocols, and obtaining both oral and written informed consent from the parents/caregivers before inclusion. The study was registered at clinicaltrials.gov (NCT00624689, February 27, 2008), and was conducted in accordance with the Declaration of Helsinki and Good Clinical Practice guidelines.

### Study formula

SF infants consumed BabySemp1 (Semper AB, Sundbyberg, Sweden) and EF infants consumed a formula modified from BabySemp1 with the addition of bovine MFGM-enriched whey protein concentrate (Lacprodan® MFGM-10; Arla Foods Ingredients, Viby J, Denmark) to account for 4% of the total protein in the EF. The macronutrient composition of the EF and SF has been described previously and also available in SI Table 3.

### Estimation of nutrient intake

3-day food diary was recorded by parents every month over 3 consecutive days at 2, 3, 4, 5, 6 and 12 months of age. All food and drink that a child consumed were recorded either by weight (grams) or volume (milliliters, deciliters, tablespoons or teaspoons) by the parents using household measures and kitchen utensils. Taste portions were noted as if the intake volume was <15 mL. Brand name and manufacturer name were recorded if the food/drink consumed was a commercial product. The nutrient intake of each complementary food was calculated according to the Swedish National Food Agency Database and food labels. Use of medications, vitamins or dietary supplementations was documented. Persistence of breastfeeding was reported but the volume was not.

### Stool collection procedure

Before or at the time of each visit, stool samples were collected by parents into a plastic container. The samples were immediately frozen at −80 °C or, if the sample was collected at home, frozen at −20 °C in a home freezer before transportation to the clinic and storage at −80 °C. The maximum storage time at −20 °C was 3 days.

### Fecal metabolite extraction

After manual homogenization with a sterile microspatula, avoiding undigested food, approximately 250 mg of fecal material were weighted and then extracted by vigorously vortexing in 1.5 ml ice-cold Dulbecco’s phosphate buffered saline (DPBS, 1X, pH 7.4) followed by centrifugation. The pellet was collected, frozen at −80 °C, and subsequently lyophilized to determine fecal dry weight (Labconco FreeZone 4.5 L Freeze Dry System, Labconco, Kansas city, MO). The supernatants were carefully collected and sequentially filtered through a syringe filter with 0.22 µm pore size (Millex-GP syringe filter, Millipore, Billerica, MA) and an ultracentrifugal filter with a 3k Da molecular weight cut-off (Amicon ultra centrifugal filter, Millipore, Billerica, MA) to remove microbes and excess proteins. 23 µl of internal standard (DSS-d6 in 5 mM, with 0.2% sodium azide in 99.8% D_2_O) was added to 207 µL filtrate. The pH of each sample was adjusted to 6.8 ± 0.1 by adding small amounts of NaOH and HCl to minimize pH-based peak movement. 180 µL aliquots were subsequently transferred to 3 mm Bruker NMR tubes (Bruker, Brillerica, MA) and stored at 4 °C until spectral acquisition.

### NMR acquisition, data processing and quantification

^1^H NMR spectra were acquired at 298K using the NOESY ^1^H presaturation experiment (‘noesypr1d’) on Bruker Avance 600 MHz NMR spectrometer (Bruker BioSpin, Germany). Spectral acquisition and processing parameters are identical to our previous work from this cohort^13^. Spectra were manually processed and profiled using Chenomx NMR Suite v8.3 (Chenomx Inc, Edmonton, Alberta, Canada). The concentration of fecal metabolites were estimated as:

$${\rm{O}}utput\,from\,Chenomx\times \frac{230}{207}\times \frac{fecal\,water\,estimate+1500}{fecal\,water\,estimate},$$

where fecal water estimate is wet weight (mg) − dry weight (mg).

### Stool water % and bristol score

Stool water % was calculated as (wet − dry)/wet stool weight * 100. Bristol stool score^92^, a scale from category 1 to 7 that indicates from hard to watery stool, was documented by one laboratory technician to ensure consistency.

### Fecal microbial DNA extraction and library preparation

To minimize the freeze-thaw cycle, the fecal metabolite and microbial DNA were extracted on the same day for each sample. Approximately 250 mg of fecal material was extracted according to the HMP protocol using MoBio PowerLyzer PowerSoil DNA isolation kit (MoBio, Carlsbad, CA)^93^. The HMP protocol was modified from the manufacturer’s instruction as follows: (1) samples were heated at 65 °C for 10 min and then 95 °C for 10 min after adding C1 solution; (2) bead-beating of the samples was performed at 6.5 m/s for 1 min followed by 5 min rest, and bead-beating again for 1 min (FastPrep-24 bead beater, MP Bio, Solon OH); and (3) after bead beating, samples were centrifuged for 3 min to ensure pellets fully formed. The DNA purity was determined spectrophotometrically (NanoDrop 2000C Spectrophotometer, Thermo Fisher Scientific, Waltham, MA, USA).

The V4 region of the 16S rRNA gene was targeted using F515/R806 primers^94^ modified according to Bokulich *et al*.^95^ to contain an illumina adapter sequence on the forward primer. An 8 bp Hamming error-correcting barcode was attached to the 3′ end of the forward primer to enable sample multiplexing. PCR reactions were performed in 20 µL reaction volumes following the protocol developed by Gohl *et al*.^96^. PCR reactions contained DNA template, DMSO (Fisher Scientific, Waltham, MA), 5X KAPA HiFi Buffer (KAPA Biosystems, Woburn, MA), dNTP Mix (10 mM), KAPA HiFi HotStart Polymerase (KAPA Biosystems, Woburn, MA), PCR-grade water (MoBio, Carlsbad, CA), DNA template and primers. The ideal volume of DNA template in the PCR reaction was estimated using a dilution series experiment followed by a quality check via gel electrophoresis of the amplicon product. The PCR reaction consisted of an initial 95 °C for 5 min followed by a 25-cycle program of 20 s at 98 °C for denaturation, 15 s at 55 °C for annealing, 60 s at 72 °C for primer extension and a final extension of 72 °C for 10 min. All reactions were amplified in duplicate and combined prior to purification.

All amplified PCR products were quality checked by gel electrophoresis. The band intensity (around 380 bp) was visualized using SYBR safe DNA stain (Invitrogen) and its quantity (in ng/µL) was estimated using a molecular ladder with known concentration (BioRad EZ ladder 1 kb) through ImageLab software (v5.2.1, BioRad, Hercules, CA). Amplicons were pooled at equimolar ratio and purified using the QIAquick PCR Purification Kit (QIAGEN, Hilden, Germany) using a modified protocol from the manufacturer’s instruction. To maximize DNA yield and purity in the final library, amplicons were first combined into 12 separate libraries (not exceeding the column DNA capacity) and each were cleaned according to the manufacturer’s instruction. The final elusion was made by eluting the first column with 60 µL of elusion buffer and all subsequent column were eluted from the filtrate from the previous column. A Purified amplicon library was quality checked by Bioanalyzer and submitted to the UC Davis Genome Center DNA Technologies Core for 250 bp paired-end sequencing on the Illumina MiSeq platform.

To avoid airborne contamination during extraction as described in Salter *et al*.^97^, the DNA extraction was performed using PCR clean and sterile filter pipette tips and in a cleaned biosafety cabinet sanitized by UV for at least 30 min prior to the experiment followed by surface decontamination by DNAaway (Thermo Scientific). One negative control “blank sample” from extraction of PCR-grade water (MoBio, Carlsbad, CA) was included for each batch. This batch sample was subsequently amplified via PCR, visually inspected using gel electrophoresis and included in the final DNA library.

### Analysis of 16 s amplicon sequence

A sliding window trimming of 4 bases wide on the low quality bases (quality score <20) was performed using Trimmomatic^98^ (version 0.33) at the end of both forward and reverse sequences. About 0.5% poor quality sequences were excluded. The subsequent sequence data was analyzed using the Quantitative Insights Into Microbial Ecology (QIIME) platform^99^ (version 1.9.0). Forward and reverse reads were joined using Fastq-join^100^ via *join_paired_end.py* where at least 50 bp of overlapping was required and the maximum difference was below 5%. About 74.16% of the sequences remained after joining paired end reads. The subsequent joined sequences were preprocessed and demultiplexed according to the following criteria: (1) removal of primer sequences; (2) minimum acceptable Phred quality score Q > 20: and (3) no barcode mismatch allowed. Closed OTU picking at 97% identity was done using sortmerna^101^ against the greengenes database (greengenes 13_8)^102^. After removing singletons (0.011% of total sequences), the OTU table was normalized by the copy number using *normalize_by_copy_number.py* function developed in PICRUSt (version 1.1.3)^103^.

### Statistical analysis

Statistical computing and graphical generation were performed using the R programing environment. OTU table handling was achieved using *phyloseq* and the *microbiome* package. α-diversity and *β*-diversity distance matrices were computed using *DiversitySeq* and the *vegan* package, respectively. Generalized log transformation (defined as *log*(*y* + *sqrt*(*y*^2^ + *lambda*))) was applied to all metabolomics data where *lambda* is 1. Principal coordinate analysis was computed using the *pcoa* function from the *ape* package. Adonis, a nonparametric implementation of a permutational analysis of variance, was computed using the *adonis* function from *vegan* with permutation set at 999. The spread (homogeneity) within a group was determined as 1 - cor where cor is the average spearman correlation between samples and the overall group-wise average. This feature is implemented in the *divergence* function in the *microbiome* package.

The differential analysis for the microbiome was computed at the genus level using ANCOM^104^ and controls for False Discovery Rate (FDR). A pseudo count value of 0.001 was added for the relative abundance data. For other data, significance between groups was evaluated using the Kruskal-Wallis test (*kruskal.test* function) and *p*-values from the Kruskall-Wallis test were further adjusted by FDR (*p.adjust*(, *method* = *‘fdr’*)). The overall level of significance was set at p < 0.05.

Effect size between BF and FF and between SF and EF was evaluated using Cliff’s delta (δ) statistics using the *cliff.delta* function from *effsize* package. The 95% confidence interval of each computed Cliff’s delta was estimated assuming a normal distribution (*use.normal* = *TRUE*). The magnitude was assessed using the thresholds suggested by Romano *et al*.^105^, where |δ| < 0.147 corresponds to negligible, |δ| < 0.33 corresponds to small, |δ| < 0.474 corresponds to medium, otherwise large.

The square of Pearson’s correction coefficient (r^2^) was computed using *cor*, (*method* = *“pearson”*) to evaluate the strength of the correlation. All plots were generated using *ggplot2*.

## Supplementary information

CONSORT Checklist

Study Protocol

Supplementary Information

## Data Availability

16S sequencing data from this study was deposited in the European Nucleotide Archive (accession code ERP112481) and Qiita^106^ (study ID: 12021). NMR spectra and relevant metadata are available from the corresponding author on request.
