# Supplementary material for: Fecal microbiome and metabolome of infants fed bovine MFGM supplemented formula or standard formula with breast-fed infants as reference: a randomized controlled trial
Source: Sci Rep. 2019 Aug 12;9:11589. doi: 10.1038/s41598-019-47953-4 (PMC6690946; doi:10.1038/s41598-019-47953-4)
Supplement: Supplementary file 2 — Study Protocol [file 41598_2019_47953_MOESM2_ESM.pdf]

Growth and development with low-energy low-protein MFGM-supplemented infant formula:

ClinicalTrials.gov identifier: NCT00624689

Sponsors:

Swedish Innovation Agency (VINNOVA)

Västerbotten City Council

Semper/Hero

Investigators:

Dr. Niklas Timby, Dr. Magnus Domellöf, Dr. Olle Hernell

Department of Clinical Sciences, Pediatrics, Umeå University, SE 901 85 Umeå, Sweden

Phone: +46907852103

Dr. Carolyn Slupsky, Dr. Bo Lönnerdal

Department of Nutrition, University of California Davis

Davis, California. 95616. USA

Phone: 530-219-5757;

Clinical Laboratories involved in the research:

Pediatric lab, Department of Clinical Sciences, Pediatrics, Umeå University, SE 901 85 Umeå, Sweden

Department of Nutrition, University of California Davis, Davis, California. 95616. USA

Project Summary (300 words)

Rationale

Today, there is strong scientific support for the theory that nutrition in utero and during the first 3 years of life influences growth and health outcomes later in life. Formula-fed infants have disadvantages compared to breast-fed infants. Formula-fed children have higher risk of obesity and cardiovascular disease in adult life. There are also differences in infectious diseases and neurodevelopment. All these differences can potentially be explained by differences between the content of formula and human milk.

Objectives

Our research group has designed a new infant formula which is unique in the world today. It has lower energy density and lower protein content compared to standard formula and is supplemented with biologically active factors, MFGM (Milk Fat Globule Membrane). The objectives of the study were to explore the effects of the modified formula on growth, development, acute illness and future health problems.

Methods

In a randomized controlled trial we will compare children fed a modified formula to children fed a standard formula and to a breast-fed reference group with close follow up of growth, metabolic factors, neurodevelopment and illness until 6.5 years of age.

Populations

160 exclusively formula-fed healthy term infants and 80 exclusively breast-fed healthy term infants are recruited before the age of 2 months.

Timeframe

2008	Start recruiting
2011	Recruiting completed
2012	12 months follow-up completed
2018	6.5 years follow-up completed

Expected outcomes

Our hypothesis is that infants fed the modified formula, compared with infants fed a standard formula, will have a development more similar to a breast-fed reference group regarding growth, psychomotor development, infectious morbidity and metabolic profile including fat metabolism, protein metabolism, oral microbiota, fecal microbiota and fecal metabolome.

Rationale & background

There is currently strong support for the hypothesis that nutrition and growth during early infancy influences the risk of atherosclerosis, type 2 diabetes and coronary heart disease in adult life [1-3]. Cardiovascular disease is globally one of the major public health problems and the incidence is increasing [4]. According to ”the growth acceleration hypothesis”, rapid growth during infancy increases the risk of obesity, type 2 diabetes and coronary heart disease in adult life due to programming effects on metabolic systems [3, 5].

Formula-fed infants are at risk since they have a more rapid weight gain compared to breast-fed infants [6, 7] and they have also been shown to have 39% higher risk of type 2 diabetes [8], 13% higher risk of obesity [9] and 1.1 mm Hg higher systolic blood pressure [10]. Such a small difference in blood pressure may be unimportant on an individual basis but it has been shown that even small differences in risk factors on a population level can give large health effects in the community. For example, reducing the diastolic blood pressure by 2 mmHg in the population, would reduce the incidence of ischaemic heart disease by 6% and the incidence of stroke by 15% [11].

There are likely many factors contributing to the increased risk of later health problems for formula-fed infants. They have 55-80 % higher protein intake than breast-fed infants [12]. Higher protein intake leads to higher levels of growth-promoting hormones like insulin and IGF-1 and a more rapid growth [13]. We have, in a recent cohort study, found that a high protein intake at 1½ and 4 years is the dietary factor that corresponds best to overweight and obesity at 4 years (unpublished data). Another factor could be that formula feeding, compared to breast-feeding, impairs the infant’s self-regulation of food intake [14]. Reduced self-regulation and increased maternal control of food intake has been shown to accelerate growth in overweight children [15].

Formula-fed infants have higher risk of respiratory tract infections [16], otitis [17, 18] and gastroenteritis [19] compared to breast-fed infants. One possible explanation is that breast milk contains antibacterial, antiviral and immunomodulating factors that are not present in formula. Some of these are unique to human milk [20], others are present in cow’s milk but are inactivated during processing to infant formula.

Several large studies have shown better psychomotor development and cognitive function in breast-fed compared to formula-fed infants. In a meta-analysis of studies adjusting for potential confounders (socioeconomic, educational and hereditary factors), a difference of 3.16 IQ-points was found at 6-23 months of age to the breast-fed children´s advantage persisting up to 15 years of age [21]. Due to ethical and practical problems, randomized studies of breast-feeding vs formula-feeding are virtually impossible to perform. However, one single randomised study from the 80’s showed higher developmental scores in breast milk-fed preterm babies supporting the hypothesis that there are factors in breast milk that support neural development [22]. In a recent study in Belarus where the rate of breast-feeding was low, hospitals were cluster randomized to breast-feeding promotion or no intervention. The breast-feeding promotion significantly increased the rate of breast-feeding and was associated with 7.5 points higher verbal IQ at 6.5 years [23].

This difference in cognitive development could be due to the presence of factors in breast milk supporting the development of the nervous system, factors not present in current infant formulas. Long-chain polyunsaturated fatty acids (LCPUFA) have been suggested to play a role in neural development, and may be beneficial to preterm infants. A recent Cochrane review, however, found no beneficial effects of supplementation LCPUFA on physical, visual or developmental outcomes in term infants [24]. Recently, several other factors besides LCPUFA have been suggested to play a role in neural development and explain some of the differences between breast-fed and formula-fed infants. Sphingomyelin is the major component of the phospholipid fraction in breast milk and the concentration is much lower in formula. Sphingomyelin is metabolised to ceramide which is essential for nervous system myelination. In a rat model, oral sphingomyelin increased myelination [25]. The concentration of choline differs between breast milk and formula; the phosphocholine concentration being higher in breast milk. In a rat model, supplementation with choline improved memory and learning [26]. Sialic acid is a monosacharide which is present in high concentrations in human brain and in human milk, and has been suggested as a potential breast milk factor affecting the neural system [27]. In a pig model, sialic acid supplementation improved memory and learning [28].

Most of these biologically active factors with potential to improve neurodevelopment are present in a recently characterized milk fraction: the milk fat globule membrane (MFGM), which is the phospholipid layer surrounding the fat droplets in human and bovine milk. With new dairy industry techniques, it is now possible to purify the MFGM-fraction from bovine milk. Phospholipids with potential effects on the neural system make up 30% of the fat in MFGM and sphingomyelin, phosphatidylcholine and phosphatidylethanolamine are the dominating phospholipids [29]. This makes MFGM a potentially very important component in breast milk that can be added to an infant formula.

In addition, MFGM has been suggested to have anti-infective properties. Butyrophilin, MUC1 and PAS6/7 (lactadherin), CD14, toll-like receptor 1 and 4 are proteins in MFGM with antimicrobial effect [29, 30]. MFGM supplementation to complementary food to 6-11 months old children gave a reduction of diarrhea in a recent study in Peru [31].

Study goals and objectives:

The aim of the TUMME-study is to, by changing the content of infant formula, reduce observed differences between formula-fed and breast-fed infants with respect to acute and future health as well as neurodevelopment. This is made by using new knowledge in nutritional and dairy sciences together with state-of-the art analysis of health outcomes at follow-up.

Hypothesis

We have two main hypotheses: 1) an infant formula with lower energy density and lower protein content reduces the risk of obesity and risk for cardiovascular disease and 2) supplementation of infant formula with MFGM will improve neurological development and decrease the risk of infections.

Study Design

The study design is a randomized double-blinded intervention study of modified versus standard infant formula with exclusively breast-fed infants serving as reference. Infants are included before two months of age and the intervention lasts until 6 months of age. Inclusion criteria are gestational age at birth 37-42 weeks, birth weight 2500-4500 grams, absence of chronic disease and parental intention to exclusively feed infant formula or exclusively breastfeed during the intervention period. Follow-up will continue until 6.5 years of age.

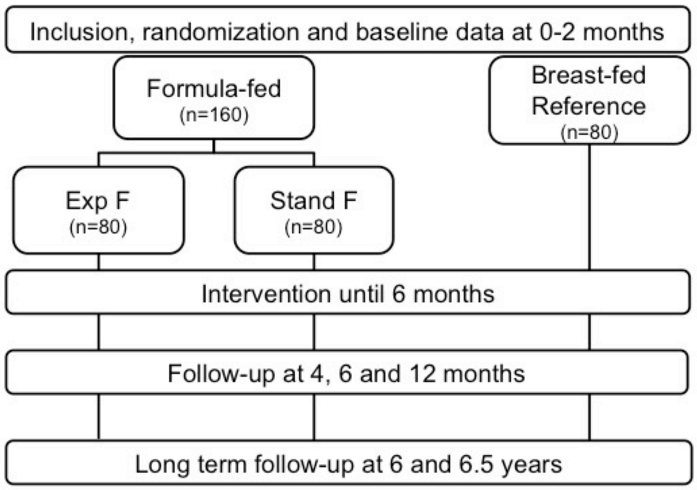

Figure. *Flow chart of the study.*

Methodology

Interventions including process of randomization and blinding, description of stopping roles, procedures and conditions for breaking the codes

Intervention

Recommendations from European Society for Pediatric Gastroenterology, Hepatology and Nutrition (ESPGHAN) and the directives of the European Union says that the energy density in infant formula should be between 60-70 kcal/100 ml. Our modified formula contains 60 kcal/100 ml compared to 66 kcal/100 ml in standard formula. The protein content is 1,20 g/100ml compared to 1,27 g/100ml. If the ingested volume is not affected by the change in composition, this means that a child that typically eats 750 ml/day of formula would ingest 450 kcal/day with modified formula instead of 495 kcal/day with standard formula and 9,0 g protein/day instead of 9,5 g protein/day, respectively. Thereby we have pushed the energy and protein levels as close to the present lower limits as possible and closer to the situation of the breast-fed child. Our modified formula is also supplemented with the MFGM fraction described above with biologically active components that makes the formula even more similar to breast milk.

Measurements, observations and laboratory analyses

We follow anthropometry (length, weight, head circumference and knee-heel). To further assess growth, we also analyse IGF-1 and body composition. Body composition is assessed with air-displacement plethysmography (PeaPod). The fecal microbiota develops during the first months of life and has recently been suggested as an important risk factor for obesity [32, 33]. We follow the fecal microbiota and fecal metabolome from inclusion until 12 months of age. The modified formula has a lower protein content and we measure urea (BUN) and amino acids in plasma to assess the impact of the diet on metabolism. We use a validated instrument (Child Feeding Questionnaire) to measure parental beliefs, attitudes and practices regarding child feeding, with a focus on obesity proneness [34]. Food intake is documented by the parents using a 3-day food diary at 2, 3, 4, 5, 6 and 12 months of age. Hypertension, high LDL:HDL ratio and high apoA1:apoB ratio are well-known risk factors for cardiovascular disease. We follow blood pressure and fasting lipid profile (total and LDL/HDL cholesterol and apo-lipoprotein A-1 and B). Homocysteine and highly-sensitive CRP are both known pediatric precursors of atherosclerosis [35, 36]. CRP is a marker for low-grade inflammation and to further investigate inflammation, we also analyse fecal calprotectin. An increased intima and media thickness (IMT) of the vessel wall is an early sign of development of atherosclerosis [36]. We measure IMT of the carotid artery with ultrasound. We also measure leptin:adiponectin ratio that has been shown to be an independent predictor of IMT and possibly cardiovascular disease [37]. To assess insulin resistance, fasting blood glucose and insulin are measured and homeostasis model assessment insulin resistance (HOMA-IR) is calculated [38]. Developmental testing is performed by a psychologist at 12 months and 6.5 years with the age-appropriate tests Bayley Scales of Infant and Toddler Development-III and Wechsler Intelligence Scale for Children (WISC), respectively. Visual acuity is also a marker for neurodevelopment and measured using sweep visual evoked potential (s-VEP), an EEG-based test that makes it possible to test visual acuity before the child is able to follow instructions. S-

VEP is the golden standard for assessing visual acuity in infants [39]. We measure sialic acid in saliva and a fecal marker for sphingomyelin, two factors with potential to enhance neurodevelopment [25, 27, 28]. Fatty acid composition of erythrocyte membranes are measured as an outcome to assess the change in phospholipids derived from the MFGM fraction and with possible impact for brain development. All symptoms of illness and medical visits are documented by the parents in a daily diary until 1 year of age.

	Start	4 mo	6 mo	12 mo	6 - 6.5 yr
Anthropometry	X	X	X	X	X
IGF-1	X	X	X	X	X
Body composition	X	X			
Fecal microbiota and metabolome	X	X	X	X	
Urea (BUN), amino acids in plasma	X		X	X	
Child Feeding Questionnaire		X		X	
Blood pressure	X	X	X	X	X
Fasting lipid profile	X		X	X	X
Homocystein in plasma	X	X	X		
High sensitive CRP	X	X	X	X	X
Calprotectin in faeces	X	X	X		
Intima media thickness of carotid artery			X		X
Leptin, adiponectin	X	X	X	X	X
Fasting insulin and blood glucose	X	X	X	X	X
Developmental testing by psychologist				X	X
S-VEP			X		
Sialic acid in saliva	X	X			
Marker for sphingomyelin in faeces	X		X	X	X
Fatty acid composition of erythrocyte membranes	X		X		

Table. *Summary of measurements.*

Safety Considerations

The composition of both formulas used in the study are within the current recommendations and directives. We have not identified any risk for the study persons for adverse events from the intervention or from measurements. Any medical problems will be referred to standard medical care.

Follow-Up

Children are followed with study visits from inclusion until 6.5 years of age, 6 years after finishing the intervention.

Data Management and Statistical Analysis

We aim to find a difference of 0.5 SD for each main outcome variable, corresponding to a difference of 0.4 kg in weight at 6 months and 7.5 IQ points in psychomotor development. With a statistical power of 80% and a level of significance of 0.05, we need a sample size of 60 children in each group. With a drop out frequency similar to our previous studies (approximately 25%), recruitment of 80 children in each group will be sufficient. Statistical analysis will be performed with standard methods for each outcome variable.

Quality Assurance

The study follows the principles of good clinical practice (GCP). Since the intervention in this study is a diet and not a pharmaceutical agent, regulations regarding pharmaceutical studies is not applicable

Expected outcomes of the study

Commercial infant formulas have improved immensely since they were first introduced in the 1950's, and there is no doubt that children who cannot be breast-fed can be adequately nourished with formula. However, there are still disadvantages for formula-fed children and substantial health benefits may be achieved by improving infant formulas so that differences between breast-fed and formula-fed infants are further reduced. One clear difference is a more accelerated growth during the first year in formula-fed children, which seem to be a risk factor for cardiovascular disease in adult life. Another difference is cognitive development. In a global perspective, a reduced risk for infectious diseases would be very important.

Human milk is the prototype for a functional food having both short and long term health consequences. Our modified formula with low energy and low protein content together with supplementation with MFGM is unique, and intended to be more functional and more similar to human milk than current infant formulas. The combination of reduced energy density and reduced protein concentration has not been studied previously. The energy and protein contents of the experimental formula are within the current directive, albeit at the minimum level. Supplementation with MFGM to infant formula has not previously been studied. Our modified infant formula may therefore improve cognitive development and long-term health.

Ethics

The study was approved by the Regional Ethical Review Board in Umeå, Sweden (Dnr 07-083M), and will be conducted according to the principles in the Declaration of Helsinki. Written informed consent will be obtained from all caregivers. The trial was registered at clinicaltrials.gov as NCT00624689.

Informed consent forms

Original Swedish version

Bästa föräldrar!

Vi genomför en studie som syftar till att ytterligare förbättra modersmjölksersättning. Av olika anledningar kan inte alla barn ammas, och dessa barn behöver då få modersmjölksersättning. Den modersmjölksersättning som finns idag är bra och barnet får på ett säkert sätt i sig den näring som behövs. När man jämför stora grupper av barn som fått modersmjölksersättning med barn som fått bröstmjölk kan man dock fortfarande se små skillnader. Den tydligaste skillnaden gäller barnets tillväxt där barn som fått modersmjölksersättning har en liten ökad risk att få övervikt och fetma. En annan skillnad är att barn som får modersmjölkersättning har en ökad risk för vissa infektioner, något man framförallt ser i utvecklingsländer.

Dessa skillnader är bakgrunden till att bröstmjölk är den rekommenderade näringen under barnets första 6 månader och till att vi vill genomföra TUMME-studien. Vi studerar en ny sorts modersmjölksersättning som dels innehåller mindre energi än den nuvarande ersättningen, och dels innehåller ämnen som är biologiskt aktiva (medverkar tex i immunförsvaret). Dessa ämnen finns naturligt i både bröstmjölk och komjölk men har tidigare förstörts under processen när man tillverkar modersmjölksersättning. Med nya tekniker kan man nu tillverka modersmjölksersättning som innehåller dessa ämnen. Vi tror att gruppen barn som får den nya ersättningen kommer att skilja sig mindre från gruppen bröstmjölksuppfödda barn jämfört med gruppen barn som får den nuvarande ersättningen.

Studien

- Vi vill studera friska barn som har en födelsevikt mellan 2500 och 4500 gram. Dels barn som ammas helt till 6 månaders ålder, dels barn som av någon anledning inte får bröstmjölk mellan 2 och 6 månaders ålder. Barnen kommer att delas in i 3 grupper, en grupp med barn som ammas och två grupper med barn som får modersmjölksersättning. Barnen som får modersmjölksersättning kommer slumpvis att delas in i 2 grupper, en grupp som får vanlig modersmjölksersättning (BabySemp), och en grupp som får den nya modersmjölksersättningen. Modersmjölksersättningen kommer att skickas till de föräldrar som ger modersmjölksersättning och vara kodmärkt så att varken föräldrar eller studiepersonal vet vilket barn som får vilken modersmjölksersättning.

- Barnen i alla grupper kommer att undersökas enligt samma schema. Vi kommer att undersöka Ert barn vid studiens start (vid 0-2 månaders ålder), vid 4, 6 och 12 månaders ålder samt vid 6 års ålder. Vid varje besök mäts längd, vikt, huvudomfång, blodtryck och vi tar ett blodprov för att titta bland annat på blodfetter, blodsocker och inflammationstecken. Blodprov tas av en barnsjuksköterska som är mycket van vid att ta prover på spädbarn. Före sticket bedövas huden med EMLA-kräm. Vid besöken upp till 12 månaders ålder kommer vi även att ta ett avföringsprov för att bla titta på tarmbakterier och salivprov för att mäta sialinsyra.

- Varje månad upp till 6 månaders ålder kommer Ni, under en tredagarsperiod, att få fylla i en matdagbok så att vi får veta vad och hur mycket barnet brukar äta (bröstmjölk, mjölkersättning och/eller annan mat). Upp till 6 månaders ålder vill vi också att ni bokför barnets avföringar dagligen och att ni upp till 12 månaders ålder noterar när barnet har sjukdomssymptom som feber, snuva, hosta, kräkningar eller diarré i en symptomdagbok. Vid 4 och 12 månaders ålder kommer Ni att få fylla i ett kort formulär om tankar kring barnets matintag.

- Vid första besöket samt vid 4 månaders ålder kommer vi att göra en mätning av barnets kroppssammansättning med hjälp av en apparat (PeaPod) som mäter hur stor del av barnets vikt som består av fett, muskler, skelett etc. Undersökningen är helt smärtfri och ofarlig och innebär ingen röntgenstrålning för barnet.

- Vid besöket vid 6 månaders ålder kommer vi att göra en mätning av synskärpa (SWEEP-VEP) där barnet får sitta i en förälders knä och titta på en monitor som visar streck av olika dimension. Med hjälp av fasttejpade elektroder på huvudet kan vi få ett mått på barnets synskärpa. Denna undersökning är också smärtfri och ofarlig.

- Vid 6 månaders ålder kommer vi även att göra en undersökning av barnets blodkärl på halsen och i buken med ultraljud för att mäta tjockleken av blodkärlsväggen. Ultraljud är smärtfritt och ofarligt.

- Vid 12 månaders ålder kommer vi att göra en bedömning av barnets utveckling genom att låta dem utföra vissa enklare uppgifter.

- Vi kommer även att vilja följa upp barnet för att se hur det mår när det har blivit ännu större. Därför kommer Ni att kallas för en undersökning när barnet är 5 år. Då mäter vi barnets vikt, längd och blodtryck, tar ett blodprov och vi kommer även att utföra en bedömning av barnets utveckling. Vid 5 års ålder kommer vi att ånyo göra en ultraljudsundersökning av blodkärlen på halsen och i buken.

Deltagande

Ni som deltar i studien kommer att ha ständig möjlighet till telefonkontakt med våra barnsjuksköterskor som jobbar med studien.

Deltagandet i studien är helt frivilligt och kan när som helst avbrytas. Övriga kontakter med hälso- och sjukvården, som t.ex. besök på BVC eller akuten, påverkas inte vare sig av om Ni väljer att vara med, avstå eller avbryta Ert deltagande i denna studie.

Alla resultat kommer att behandlas så att obehörig inte kan ta del av dem. Prover kommer att lagras i biobank vid enheten för pediatrik, Umeå Universitet. De märks med en kod så att de inte kan kopplas samman till en individ. Proverna får inte användas för annat än denna studie. Om det i framtiden skulle bli aktuellt att använda proverna i en annan studie måste det i så fall godkännas av forskningsetisk kommitté och Ni kan då komma att kontaktas igen. Vid sammanställningen av resultaten kommer alla uppgifter att vara avidentifierade och redovisning/publicering av data kommer att ske så att inget barns identitet eller enskilda provresultat kan utläsas. Ansvarig för barnets personuppgifter är Umeå Universitet. Enligt personuppgiftslagen har Ni rätt att ta del av de uppgifter som lagrats om Ert barn, detta får Ni genom att kontakta någon av de ansvariga för studien.

Vill Ni ha ytterligare information, är Ni välkomna att vända Er till någon av oss.

Translated to English

Dear Parents,

We are conducting a study with the aim to improve infant formula. For different reasons, breast milk is not available for all infants and these infants need formula. Current formulas available in the market are good and give the infant a safe nutrition. However, when comparing large groups of infants there are small differences between formula-fed and breast-fed infants. The most prominent difference is the growth, and formula-fed infants have a small over-risk for overweight and obesity. Formula-fed infants also have a higher risk for some infections, mostly observed in developing countries.

These differences are the rationale for recommending breastfeeding during the first 6 months of life, and the reason why we want to conduct this study. We are studying a new infant formula with less energy and biologically active components (with possible effects i.e. in the defence against infections). These components are natural components of both human and cow's milk but have previously been discarded during manufacturing of infant formula. With new techniques, it is now possible to add these components to formula. Our hypothesis is that infants fed the new formula will differ less from breastfed infants compared to infants fed standard formula.

The study

- We are studying healthy infants with a birth weight between 2500- 4500 grams. We study infants that are breastfed until 6 months of age and infants whose mothers have stopped breastfeeding before 2 months of age. The infants will be divided into 3 groups: one group with breastfed infants and two groups with formula-fed infants. The formula-fed infants will be randomized into 2 groups; one group receiving standard formula (BabySemp), and one group receiving the new formula. The formulas will be sent to the parents and be marked with a code (blinded) so that neither parents nor study personnel will know which infant is fed with which formula.

- All children in the study will be examined in the same way. We will examine your child at the beginning of the study (0-2 months of age), at 4, 6 and 12 months and 6 years of age. At every visit, we will measure length, weight, head circumference, blood pressure and a blood sample will be drawn for analysis of i.e. blood lipids, blood glucose and inflammation. The blood sample will be drawn by an experienced pediatric nurse. Before the blood sample, the skin will be anesthetized with a cream. At all visits until 12 months of age, a faecal sample will also be collected for analysis of i.e. faecal microbiota and a saliva sample for analysis of sialic acid.

- Every month until 6 months of age, you will be asked to fill in a food diary during 3 consecutive days to assess the normal intake of your child. You will also be asked to fill in a diary of disease symptoms like fever, cold, coughing, vomiting or diarrhea. At

4 and 12 months you will be asked to answer a form on attitudes about your child's feeding.

- At the first and second visits, we will measure your child's body composition using a plethysmograph (PeaPod) that can assess the fat and lean mass proportions of the child's weight. The measurement does not harm the child, is not painful and is not using x-ray.

- At 6 months, a visual test (SWEEP-VEP) will be done. The child is sitting in the lap of the parent while watching a monitor with black and white stripes. With electrodes on the head, we can assess the visual acuity. This test is not painful and does not harm the child.

- At 6 months of age, we will examine the arteries on the neck using ultrasound. Ultrasound is not painful and does not harm the child.

- At 12 months of age, we will measure the psychomotor development by giving the child some simple tasks.

- When the child gets older, we will continue the follow-up. You will be asked to come to a new visit when the child is 6 years old. We will measure length, weight, blood pressure, draw a blood sample and perform a new test of the child's psychomotor development. We will also do a new ultrasound examination of the vessels on the neck.

Participation

If you chose to participate in the study, you will have the possibility of telephone contact with our pediatric nurses working with the study. The participation is voluntary and can be terminated at any time. Other contacts with the health care system will not be affected whether you chose to participate, not participate, or terminate your participation in the study. All results will be handled so that no unauthorized person can see them. Samples will be stored in a biobank at the Unit of Pediatrics, Umeå University. They will be marked with a code so they can not be associated to an individual. The samples may not be used for anything except the present study. If samples should be used for another study in the future, this must be accepted by an Ethical Review Board and you may be contacted again. When summarizing the results, all data will be separated from the identification and in presentations and publications of data no individual child or test result will be possible to identify. Umeå University is responsible for the personal information. According to the law of personal information, you have the right to take part of all information that is stored about your child, you will get this by contacting any of the responsible investigators.

If you want more information, please contact any of us.

References

1. Barker DJ. The developmental origins of insulin resistance. Horm Res. 2005;64 Suppl 3:2-7.

2. Singhal A, Cole TJ, Fewtrell M, Deanfield J, Lucas A. Is slower early growth beneficial for long-term cardiovascular health? Circulation. 2004;109(9):1108-1113.

3. Singhal A, Lucas A. Early origins of cardiovascular disease: is there a unifying hypothesis? Lancet. 2004;363(9421):1642-1645.

4. WHO. The world health report 2003: Shaping the future, Chapter 6: Neglected global epidemics: three growing threats. Geneva: WHO, 2003.

5. Waterland RA, Garza C. Potential mechanisms of metabolic imprinting that lead to chronic disease. The American journal of clinical nutrition. 1999;69(2):179-197.

6. Baird J, Poole J, Robinson S, Marriott L, Godfrey K, Cooper C, et al. Milk feeding and dietary patterns predict weight and fat gains in infancy. Paediatr Perinat Epidemiol. 2008;22(6):575-586.

7. Dewey KG. Growth characteristics of breast-fed compared to formula-fed infants. Biology of the neonate. 1998;74(2):94-105.

8. Owen CG, Martin RM, Whincup PH, Smith GD, Cook DG. Does breastfeeding influence risk of type 2 diabetes in later life? A quantitative analysis of published evidence. The American journal of clinical nutrition. 2006;84(5):1043-1054.

9. Owen CG, Martin RM, Whincup PH, Smith GD, Cook DG. Effect of infant feeding on the risk of obesity across the life course: a quantitative review of published evidence. Pediatrics. 2005;115(5):1367-1377.

10. Owen CG, Whincup PH, Gilg JA, Cook DG. Effect of breast feeding in infancy on blood pressure in later life: systematic review and meta-analysis. BMJ. 2003;327(7425):1189-1195.

11. Cook NR, Cohen J, Hebert PR, Taylor JO, Hennekens CH. Implications of small reductions in diastolic blood pressure for primary prevention. Arch Intern Med. 1995;155(7):701-709.

12. Koletzko B, Broekaert I, Demmelmair H, Franke J, Hannibal I, Oberle D, et al. Protein intake in the first year of life: a risk factor for later obesity? The E.U. childhood obesity project. Adv Exp Med Biol. 2005;569:69-79.

13. Agostoni C, Scaglioni S, Ghisleni D, Verduci E, Giovannini M, Riva E. How much protein is safe? Int J Obes (Lond). 2005;29 Suppl 2:S8-13.

14. Taveras EM, Scanlon KS, Birch L, Rifas-Shiman SL, Rich-Edwards JW, Gillman MW. Association of breastfeeding with maternal control of infant feeding at age 1 year. Pediatrics. 2004;114(5):e577-583.

15. Farrow C, Blissett J. Does maternal control during feeding moderate early infant weight gain? Pediatrics. 2004;118:e293-e298.

16. Wright AL, Holberg CJ, Martinez FD, Morgan WJ, Taussig LM. Breast feeding and lower respiratory tract illness in the first year of life. Group Health Medical Associates. BMJ. 1989;299(6705):946-949.

17. Aniansson G, Alm B, Andersson B, Hakansson A, Larsson P, Nylen O, et al. A prospective cohort study on breast-feeding and otitis media in Swedish infants. Pediatr Infect Dis J. 1994;13(3):183-188.

18. Duncan B, Ey J, Holberg CJ, Wright AL, Martinez FD, Taussig LM. Exclusive breast-feeding for at least 4 months protects against otitis media. Pediatrics. 1993;91(5):867-872.

19. Dewey KG, Heinig MJ, Nommsen-Rivers LA. Differences in morbidity between breast-fed and formula-fed infants. The Journal of pediatrics. 1995;126(5 Pt 1):696-702.

20. Naarding MA, Dirac AM, Ludwig IS, Speijer D, Lindquist S, Vestman EL, et al. Bile salt-stimulated lipase from human milk binds DC-SIGN and inhibits human immunodeficiency virus type 1 transfer to CD4+ T cells. Antimicrob Agents Chemother. 2006;50(10):3367-3374.

21. Anderson JW, Johnstone BM, Remley DT. Breast-feeding and cognitive development: a meta-analysis. The American journal of clinical nutrition. 1999;70(4):525-535.

22. Lucas A, Morley R, Cole TJ, Gore SM. A randomised multicentre study of human milk versus formula and later development in preterm infants. Archives of disease in childhood Fetal and neonatal edition. 1994;70(2):F141-146.

23. Kramer MS, Aboud F, Mironova E, Vanilovich I, Platt RW, Matush L, et al. Breastfeeding and child cognitive development: new evidence from a large randomized trial. Arch Gen Psychiatry. 2008;65(5):578-584.

24. Simmer K, Patole SK, Rao SC. Longchain polyunsaturated fatty acid supplementation in infants born at term. Cochrane Database Syst Rev. 2008(1):CD000376.

25. Oshida K, Shimizu T, Takase M, Tamura Y, Yamashiro Y. Effects of dietary sphingomyelin on central nervous system myelination in developing rats. Pediatric research. 2003;53(4):589-593.

26. Zeisel SH. Choline: an essential nutrient for humans. Nutrition. 2000;16(7-8):669-671.

27. Wang B, Brand-Miller J. The role and potential of sialic acid in human nutrition. European journal of clinical nutrition. 2003;57(11):1351-1369.

28. Wang B, Yu B, Karim M, Hu H, Sun Y, McGreevy P, et al. Dietary sialic acid supplementation improves learning and memory in piglets. The American journal of clinical nutrition. 2007;85(2):561-569.

29. Spitsberg VL. Invited review: Bovine milk fat globule membrane as a potential nutraceutical. Journal of dairy science. 2005;88(7):2289-2294.

30. Reinhardt TA, Lippolis JD. Bovine milk fat globule membrane proteome. J Dairy Res. 2006;73(4):406-416.

31. Zavaleta N, Valencia N, Graverholt G, Staudt-Kvistgaard A, Lonnerdal B. Incidence and duration of diarrhea in peruvian infants consumin complementary food with bioactive milk fat globule membrane proteins (Abstract). J Pediatr Gastroent Nutr. 2006;42:e39.

32. Mai V, Draganov PV. Recent advances and remaining gaps in our knowledge of associations between gut microbiota and human health. World J Gastroenterol. 2009;15(1):81-85.

33. Backhed F. Changes in intestinal microflora in obesity: cause or consequence? Journal of pediatric gastroenterology and nutrition. 2009;48 Suppl 2:S56-57.

34. Birch LL, Fisher JO, Grimm-Thomas K, Markey CN, Sawyer R, Johnson SL. Confirmatory factor analysis of the Child Feeding Questionnaire: a measure of parental attitudes, beliefs and practices about child feeding and obesity proneness. Appetite. 2001;36(3):201-210.

35. Franco MC, Higa EM, D'Almeida V, de Sousa FG, Sawaya AL, Fortes ZB, et al. Homocysteine and nitric oxide are related to blood pressure and vascular function in small-for-gestational-age children. Hypertension. 2007;50(2):396-402.

36. Groner JA, Joshi M, Bauer JA. Pediatric precursors of adult cardiovascular disease: noninvasive assessment of early vascular changes in children and adolescents. Pediatrics. 2006;118(4):1683-1691.

37. Norata GD, Raselli S, Grigore L, Garlaschelli K, Dozio E, Magni P, et al. Leptin:adiponectin ratio is an independent predictor of intima media thickness of the common carotid artery. Stroke. 2007;38(10):2844-2846.

38. Hirschler V, Calcagno ML, Aranda C, Maccallini G, Jadzinsky M. Can the metabolic syndrome identify children with insulin resistance? Pediatr Diabetes. 2007;8(5):272-277.

39. Lauritzen L, Jorgensen MH, Michaelsen KF. Test-retest reliability of swept visual evoked potential measurements of infant visual acuity and contrast sensitivity. Pediatric research. 2004;55(4):701-708.
