# Supplementary material for: Fecal microbiome and metabolome of infants fed bovine MFGM supplemented formula or standard formula with breast-fed infants as reference: a randomized controlled trial
Source: Sci Rep. 2019 Aug 12;9:11589. doi: 10.1038/s41598-019-47953-4 (PMC6690946; doi:10.1038/s41598-019-47953-4)

Supplementary information

Fecal microbiome and metabolome of infants fed bovine MFGM supplemented formula or standard formula with breast-fed infants as reference: a randomized controlled trial

Xuan He[1,2], Mariana Parenti[1], Tove Grip[3], Bo Lönnerdal[1], Niklas Timby[3], Magnus Domellöf[3], Olle Hernell[3], Carolyn M. Slupsky[1,2,*]

Table of contents

- **Supplementary Tables**
    - **SI Table 1.** Number of samples in each intervention group
    - **SI Table 2.** Numbers of samples from breast-fed (BF) or formula-fed infants (Standard Formula, SF; Experimental Formula, EF) in a subset depending on the complementary food intake, with either > 60 kcal of daily energy or < 60 kcal of daily energy from complementary food.
    - **SI Table 3.** Macronutrient contents of standard and experimental formula
- **Supplementary Figures**
    - **SI Fig 1.** Summary of intake.
    - **SI Fig 2.** Differences in genus-level composition of fecal microbiome between breast-fed (BF), standard formula-fed (SF) and experimental formula-fed (EF) infants.
    - **SI Fig 3.** Significantly differentiating intestinal microbes between the formula-fed infants who consumed standard formula (SF, red) and experimental formula (EF, orange).
    - **SI Fig 4.** Discrepancy of key fecal metabolites concentration from microbial utilization of carbohydrate between breastfed (BF) and formula-fed (standard formula: SF, experimental formula: EF) infants.
    - **SI Fig 5.** Fecal metabolites that are significantly different between breast-fed (green) and formula-fed (red) infants.

SI Table 1 Number of samples in each intervention group

	Breastfed				Standard formula-fed				Experimental formula-fed			
**Age of infants**	2	4	6	12	2	4	6	12	2	4	6	12
Fecal 16s rRNA amplicon data	23	25	26	22	25	26	26	24	26	24	24	23
Fecal metabolome data	17	18	25	21	26	24	24	23	27	26	26	25

SI Table 2. Numbers of samples from breast-fed (BF) or formula-fed infants (Standard Formula, SF; Experimental Formula, EF) in a subset depending on the complementary food intake, with either > 60 kcal of daily energy or < 60 kcal of daily energy from complementary food.

	Age (months)	**Without complementary food subset**			**With complementary food subset**		
		BF	SF	EF	BF	SF	EF
Fecal 16s rRNA amplicon data	4	24	22	25	1	2	1
	6	10	10	7	16	14	19
Fecal metabolome data	4	18	22	25	0	2	1
	6	11	10	7	14	14	19

SI Table 3. Macronutrient contents of standard and experimental formula

	**Standard formula**	**Experimental formula**
**Energy (kcal/L)**	660	600
**Protein (g/L)**	12.7	12
Casein (g/L)	5	3.5
Whey (g/L)	8	8.5
**Carbohydrates/lactose (g/L)**	74	60
**Lipids (g/L)**	35	35
SFAs (g/L)	13	13.5
MUFAs (g/L)	14	13.5
PUFAs (g/L)	6	6
Linoleic acids (g/L)	4.6	4.6
α-Linolenic acid (g/L)	0.7	0.7
Arachidonic acid (g/L)	0.15	0.15
DHA (mg/L)	90	90
Cholesterol (mg/L)	40	80
Phospholipids (mg/L)	300	700

SI Fig 1. Summary of intake. **a.** Intake of study formula volume (mL/d) between the two study formulas. The nonparametric effect size is computed using Cliff's Delta to compare the difference in intake volume between the Experimental Formula (EF) and Standard Formula (SF) group. **b.** Frequency of consuming complementary food was increased from 4 months to 6 months of age (left y-axis margin, barplot). Frequency of infants consuming no complementary food reduced over time (right y-axis margin, marked with blue *). **c.** Dietary energy (kcal/day) from study formula and complementary food. **d.** Composition of the average macronutrients (protein, carbohydrate and fat) of complementary food from the breast-fed (BF), EF and SF group.

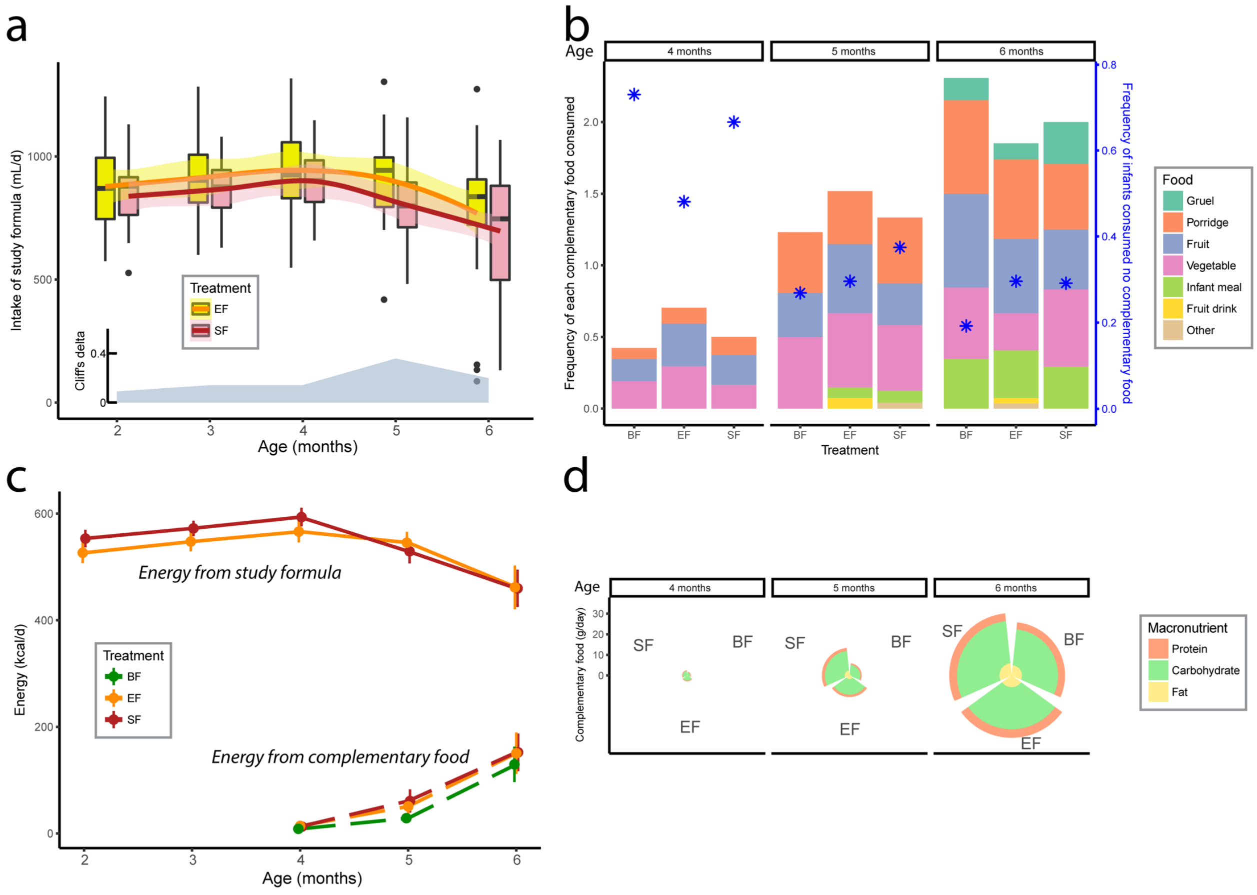

SI Fig 2. Differences in genus-level composition of fecal microbiome between breast-fed (BF), standard formula-fed (SF) and experimental formula-fed (EF) infants. Each stacked bar plot corresponds to one infant subject.

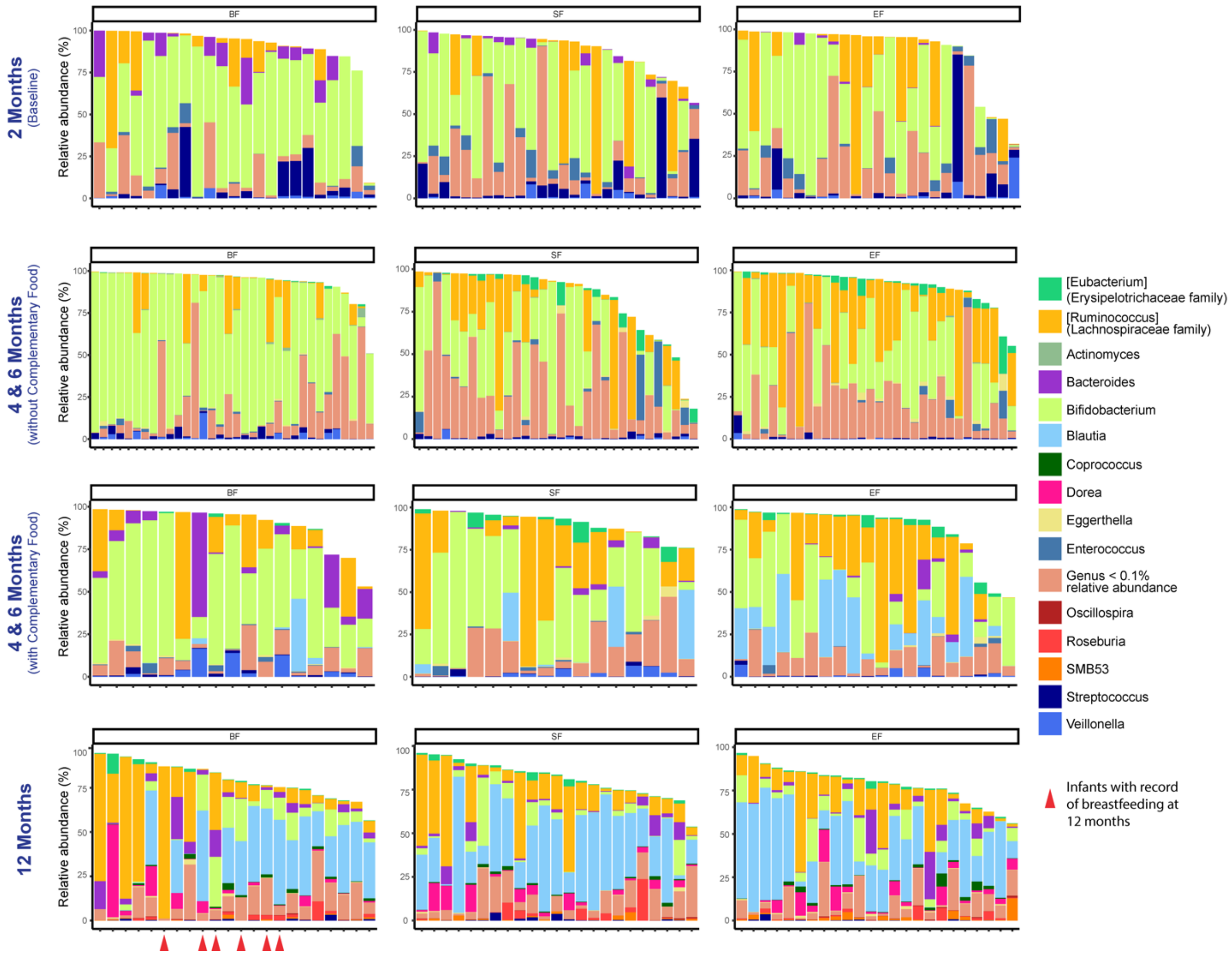

SI Fig 3. Significantly differentiating intestinal microbes between the formula-fed infants who consumed standard formula (SF, red) and experimental formula (EF, orange). **a.** Consumption of EF led to higher numbers of infants with a higher level of *Akkermansia* in the stool at 4 and 6 months of age. **b.** Infants who consumed EF had lower microbes from the *Haemophilus* genus at 12 months of age compared with those who consumed SF. The group differences were evaluated using Analysis of Composition of Microbiomes (ANCOM) followed by FDR correction at $p < 0.05$.

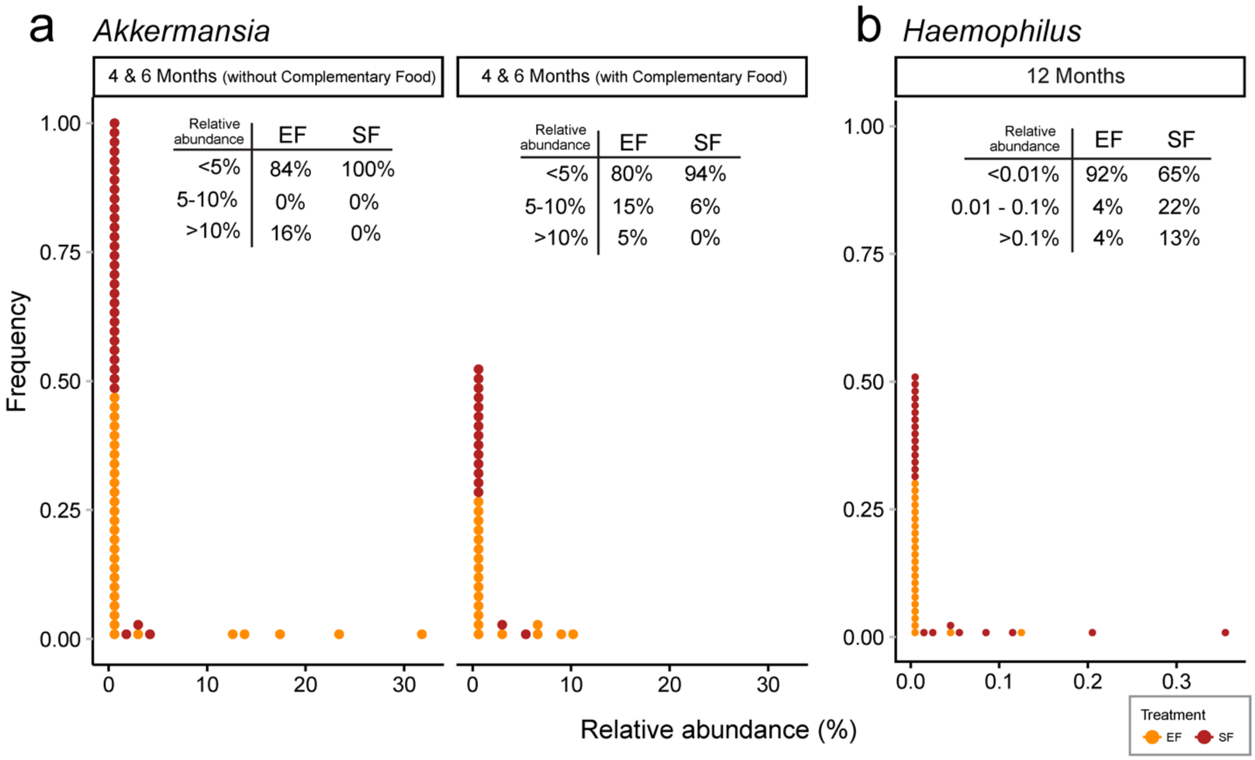

SI Fig 4. Discrepancy of key fecal metabolite concentrations from microbial utilization of carbohydrate between breast-fed (BF) and formula-fed (standard formula: SF, experimental formula: EF) infants. The sum of HMOs is estimated as the summation of 2'-fucosyllactose, 3'-fucosyllactose, 3'-sialyllactose, 6'-sialyllactose, lactodifucotetraose, lacto-N-fucopentaose I, lacto-N-fucopentaose III.

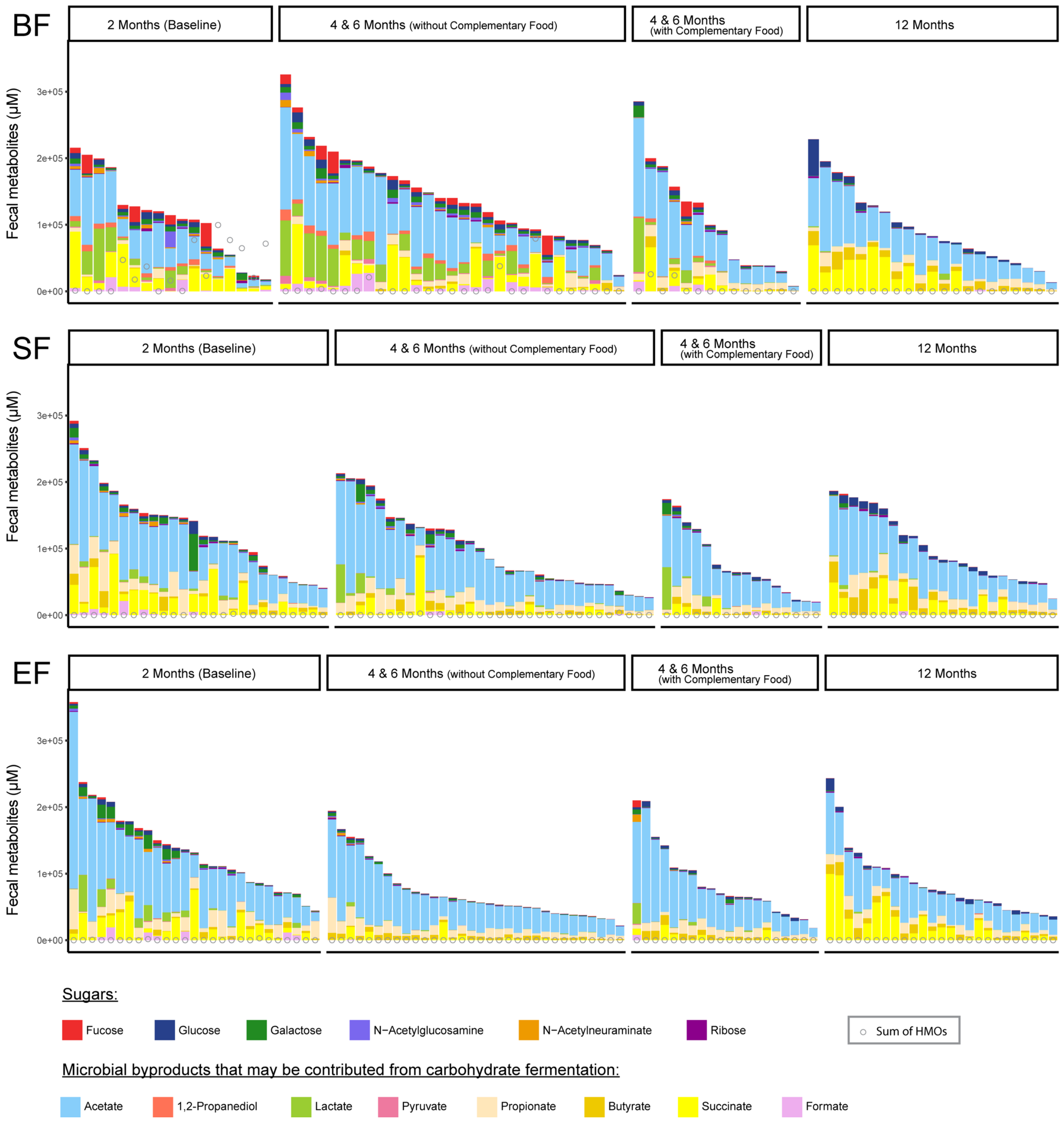

SI Fig 5. Fecal metabolites that are significantly different between breast-fed (green) and formula-fed (red) infants.

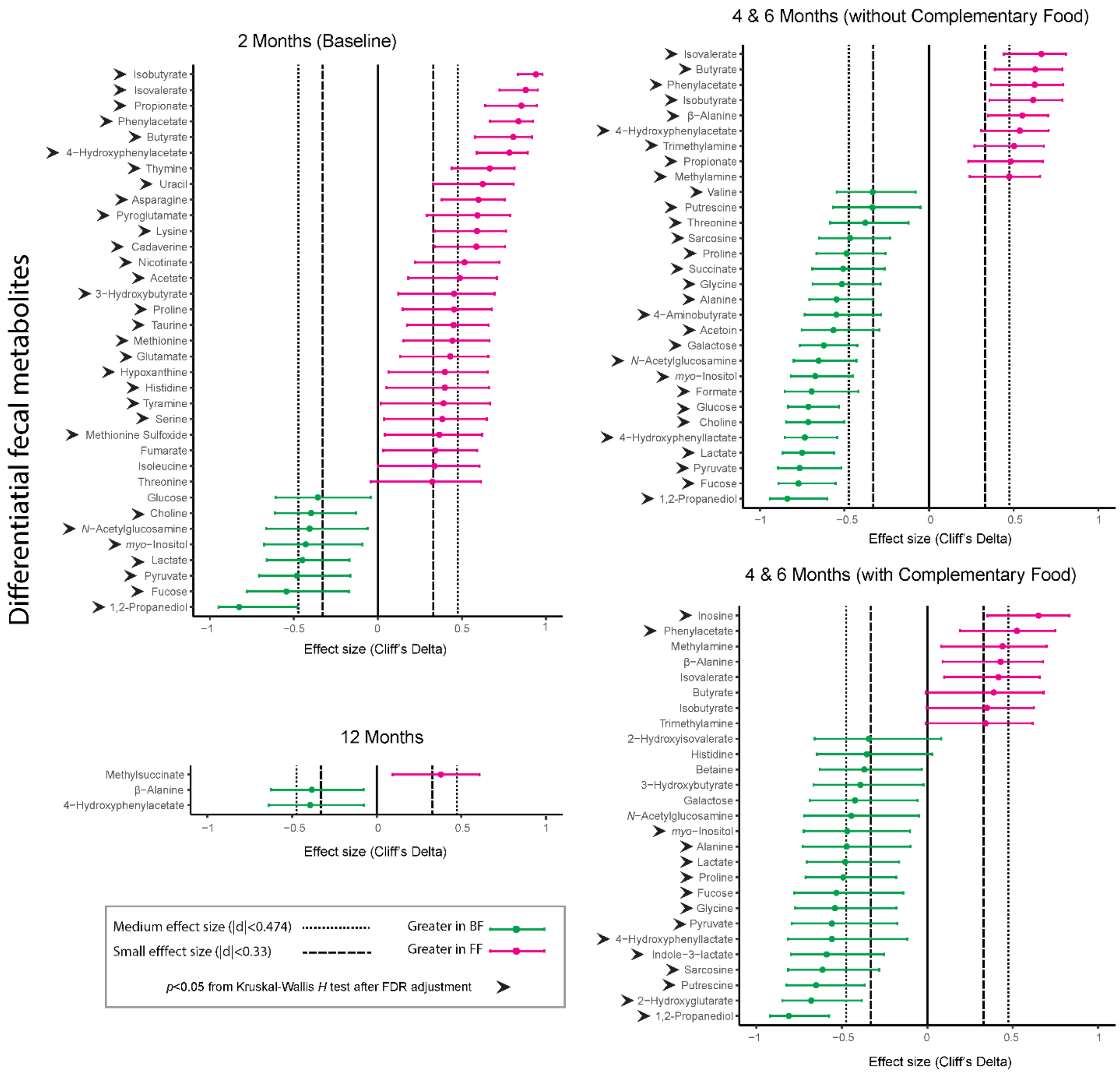

Supplement: Supplementary file 3 — Supplementary Information [file 41598_2019_47953_MOESM3_ESM.pdf]
